# Datasets of various geotechnical surveys in several arrays in the Taipei Basin

**DOI:** 10.1016/j.dib.2024.110195

**Published:** 2024-02-20

**Authors:** Chih-Chieh Lu, Yuan‑Chang Deng, Jiun-Shiang Wang, Jin-Hung Hwang, Chun-Chieh Tseng

**Affiliations:** a Division of Earth Sciences and Geotechnical Engineering, National Center for Research on Earthquake Engineering, No. 200, Sec. 3, Xinhai Rd., Taipei City 10668, Taiwan, ROC; b Department of Civil Engineering, National Central University, No. 300, Zhongda Rd., Zhongli District, Taoyuan 320317, Taiwan, ROC; c Public Works Department of Taipei City Government, No.1, City Hall Rd., Xinyi District, Taipei City 110204, Taiwan, ROC

**Keywords:** Standard penetration test, Cone penetration test, Shear wave velocity, Taipei Basin, Alluvium sites

## Abstract

The standard penetration test (SPT), seismic cone penetration test (SCPT), and various *in-situ* seismic tests are commonly utilized for geotechnical site investigations. The investigated data via these tests are widely adopted to capture site characteristics for geotechnical engineering design. However, site characterizations vary in the above *in-situ* tests, which leads to uncertainties in the corresponding engineering analysis and design. To address these variabilities, this paper meticulously carried out the above-mentioned geotechnical *in-situ* tests with rigorous supervision at 13 selected sites in the Taipei Basin, yielding several valuable datasets. The datasets consist of digital investigation data including SPT-N, soil classification, CPT-q_c_ and -fs, and the shear wave velocities (Vs) obtained from different measurements. We believe that these datasets will be beneficial for conducting various calibration studies for different geotechnical investigation methods and the corresponding geotechnical parameters.

Specifications Table

**Table:** 

Subject	Earth and Planetary Sciences
Specific subject area	Geotechnical Engineering, Geophysics
Data format	Seismic cone penetration test (SCPT) data, Standard penetration test (SPT) data (including index properties of split-samples): Raw Shear wave velocity (V_S_): Analyzed
Type of data	Table
Data collection	Except for V_S_ data collected via the multichannel analysis of surface waves (MASW) tests, all data were acquired based on the following corresponding international standards: • CPT data: ASTM D5778 (2012) • SPT data: ASTM D1586 (2018) & ASTM D4633 (2010) • V_S_ via cross-hole seismic tests: ASTM D4428/D4428M (2016) • V_S_ via down-hole seismic tests: ASTM D7400 (2019) • V_S_ via suspension PS-logging: JGS 1122 (2012) For MASW measurements, all test lines were over 60 m long, and 23 geophones (4.5 Hz) at 3 m intervals were used to receive the signals generated by an active source in each line. The SeisImager/SW software was used to derive a dispersion curve by selecting the maximum amplitudes. The deterministic inversion of dispersion curves was adopted to derive the V_S_ values according to the procedures recommended in the SeisImager manual [9].
Data source location	The dataset was obtained from alluvium sites along the Tamsui River and the Keelung River in the Taipei Basin in Taiwan. The map of these sites is shown in the manuscript.
Data accessibility	All data are available in a public repository (Mendeley Data). Data ID number: 10.17632/v7frv3k2d3.1 Direct URL to data: https://data.mendeley.com/datasets/v7frv3k2d3/1
Related research article	Wang, J.S., Hwang, J.H., Lu, C.C., Deng, Y.C. Empirical formulas for shear wave velocity prediction and their uncertainties: a case study of thirteen alluvium test sites in the Taipei Basin. Bull. Eng. Geol. Environ. 81, 450 (2022). https://doi.org/10.1007/s10064-022-02949-9

## 1 Value of the data

• Multiple prevalent *in-situ* tests, including the standard penetration test (SPT), seismic cone penetration test (SCPT), and various other seismic tests (e.g., cross-hole seismic tests and suspension PS-logging methods), were meticulously implemented at each of 13 sites in the Taipei Basin. The multiplicity of *in-situ* tests at one site benefits studies on the correlation of geotechnical parameters and calibration of their transformation variability. Moreover, the consistency studies of V_s_ measurements via different seismic tests is also enhanced by these datasets.
• The datasets contain the subsurface geo-information (reaching 30 m in depth) for each site, which provides information for soil liquefaction risk evaluation and site classification.
• This database contains two- or three-dimensional underground information for each site, which enhances studies on spatial variability and provides actual case data for numerical studies on modeling geo-formations.
• Data-centric geotechnics play an important role in response to digital transformation. However, corruption of geotechnical investigation data commonly poses a challenge. While it is time-consuming and labor-intensive to clean and organize corrupted data from legacy systems, these *in-situ* investigation records help eliminate this problem since data acquisition was supervised by doctoral fellows from the National Center for Research on Earthquake Engineering (NCREE), which ensures the quality of output results.

## 2 Background

The 2016 Meinong earthquake in Southern Taiwan highlighted the urgency of addressing soil liquefaction risks, particularly in urban settings. In response, the Taipei City Government launched the 'Home Security Plan.' This project was a concerted effort to assess and strategize soil liquefaction potential in anticipation of future seismic events. Collaborating with the NCREE as the main consultant and CECI Engineering Consultants as the executing agency, the project involved comprehensive geological surveys in over ten sites across the Taipei Basin. These surveys aimed to identify and analyze key factors in liquefaction assessment, including various testing and evaluation methodologies.

The NCREE oversaw the execution of these on-site investigations, conducted by CECI Engineering Consultants, to guarantee the data reliability and quality. The collected data are expected to significantly contribute to geotechnical research, particularly in understanding the uncertainties of simplified soil liquefaction assessment methods, shear wave velocity measurements, and correlation of different geotechnical parameters.

## 3 Data Description

**Fig. 1:**
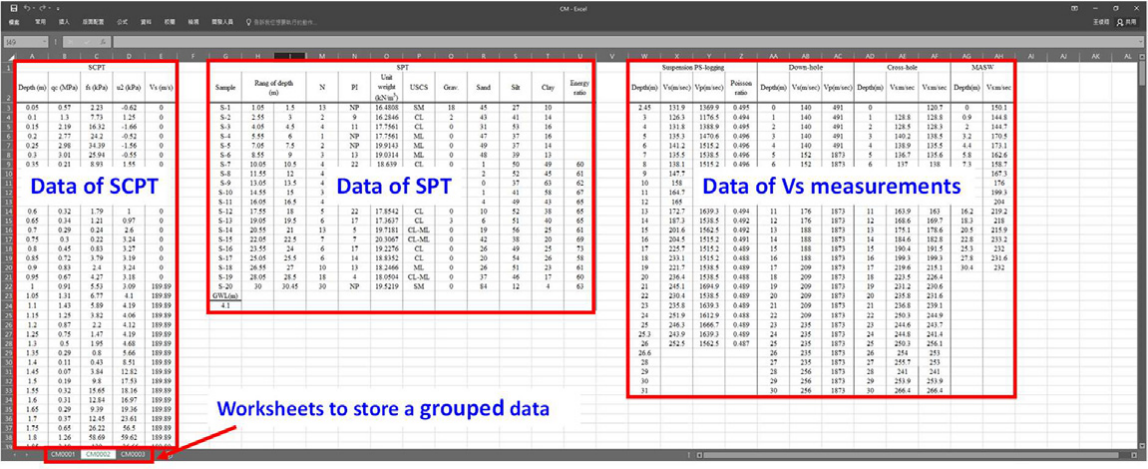
A typical example of a spreadsheet and worksheet (from sample data of site CM).

**Fig. 2:**
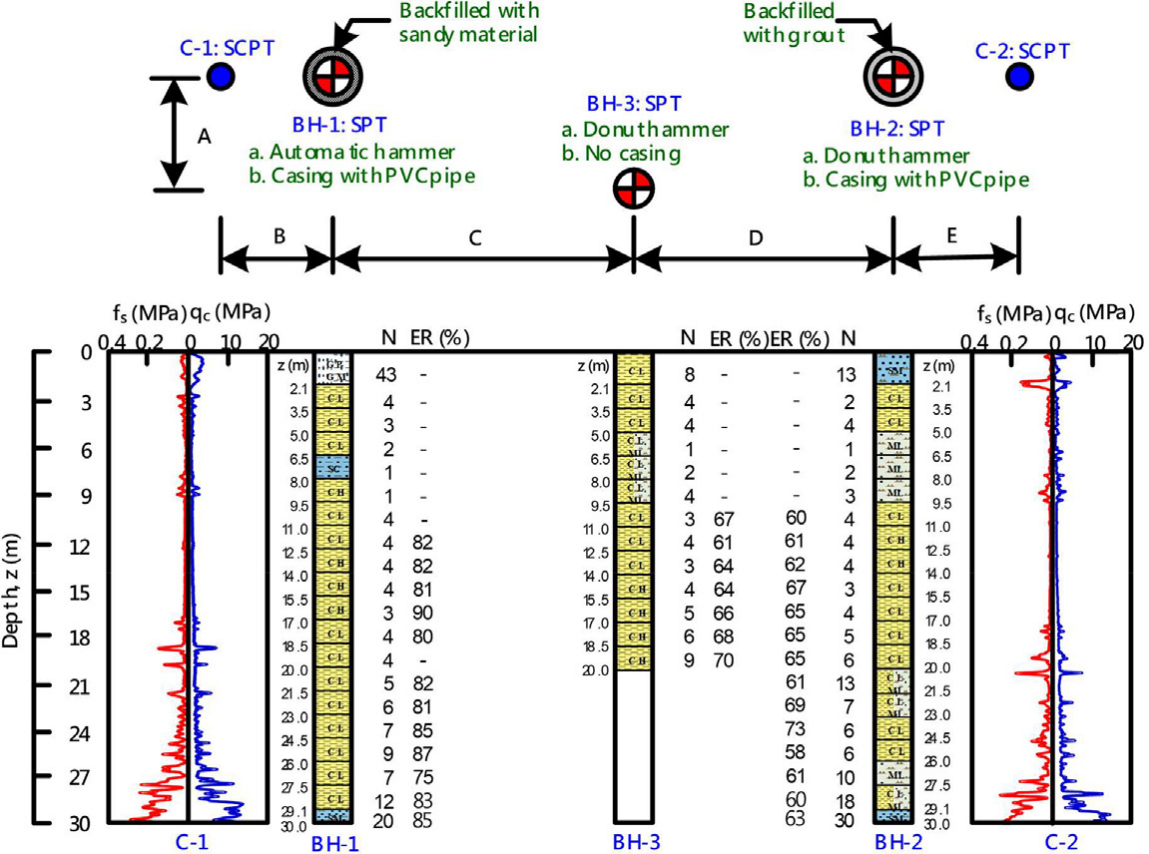
Layout and results of SPT and SCPT at site CM.

**Fig. 3:**
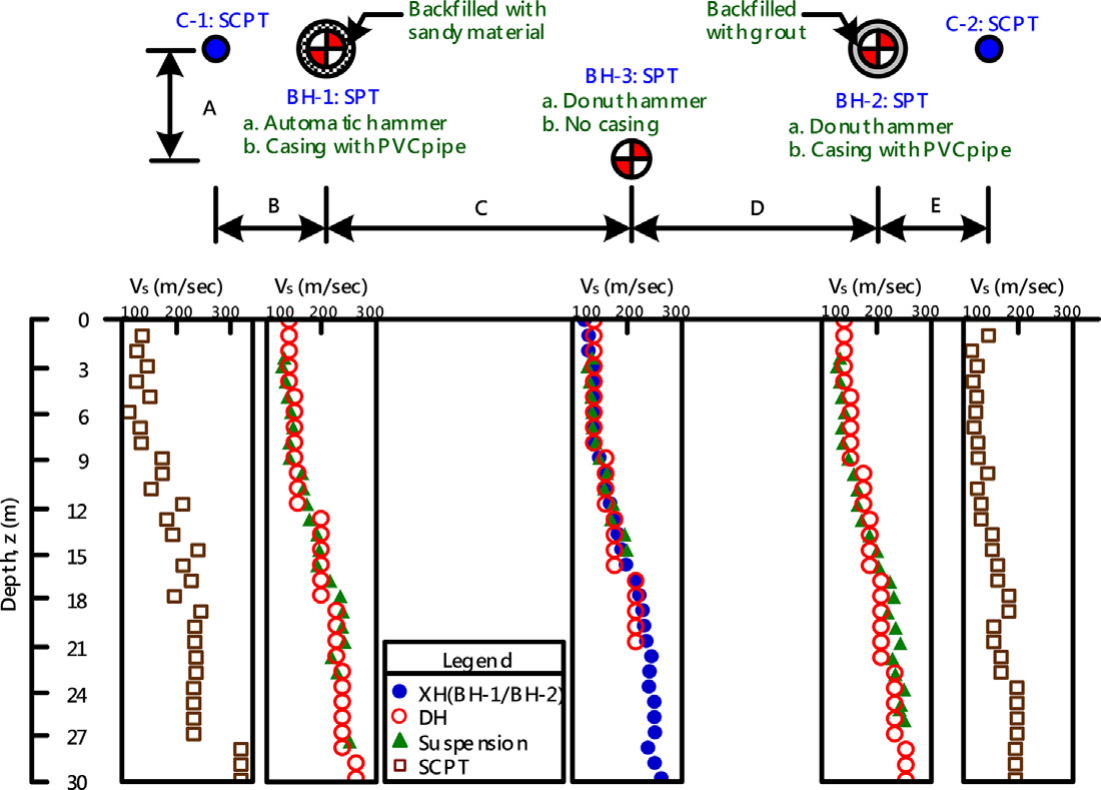
V_S_ profiles obtained using different methods at site CM.

The datasets include the data of SPTs, SCPTs, and a series of *in-situ* V_s_ measurements conducted at 13 chosen Holocene alluvium sites in the Taipei Basin. Table 1 provides a list of site labels, coordinates, and layout indices. At each of the 13 sites, two or three boreholes were drilled, and two SCPTs, traditional invasive seismic measurements (using cross-hole, down-hole, and suspension methods), and non-invasive MASW (multichannel analysis of surface waves) tests were conducted. Data from each site were compiled into a single Microsoft EXCEL spreadsheet for the 13 files. Each file comprises two or three worksheets, corresponding to the number of boreholes. Fig. 1 shows the sample data for a site in an EXCEL spreadsheet with three worksheets (three SPT boreholes). The first two characters of the worksheet title correspond to the site label given in Table 1, while the remaining characters refer to the number of the grouped data. So, please refer to Fig. 2. “0001” in the spreadsheet title refers to the data from borehole BH-1 and V_s_ measurements or soundings at or close to the borehole (i.e., at C-1). Similarly, “0002” refers to data from BH-2 and associated V_s_ measurements at or close to the borehole (i.e., C2), and “0003” to the data from BH-3 and associated V_s_ measurements. Note that some sites did not have a third borehole and therefore do not have a third worksheet. Each site has a similar plan. Fig. 2 shows the borehole and sounding layout at a typical site, providing a general plan for each location. The example in Figs. 1 and 2 demonstrates that each worksheet captures data for the SPT borehole, SCPT soundings, the V_s_ measurements near the borehole, and MASW test results. Cross-hole V_s_ measurements were repeated and appear in two worksheets when only one of the boreholes was used for cross-hole V_s_ measurements. The V_s_ measurements obtained by the MASW method cover the entire area of the site and therefore appear repeatedly throughout the worksheets. Table 2 shows the SPT data in the CM0002 worksheet and indicates sample number, testing range, SPT-N value, plasticity index (PI), unit weight, soil classification in the Unified Soil Classification System (USCS), soil content, averaged energy ratio, and ground water level during the drilling. Table 3 lists the SCPT data in the worksheet of CM0002 and contains the cone resistance (q_c_), the sleeve friction resistance (f_s_), pore pressure (u), and shear wave velocity (V_s_). Note that the shear wave velocity was only measured every 1 m, so the accuracy is not as high as that for other outputs. Fig. 2 shows the profiles of the SPT boreholes and SCPT soundings at site CM as an example. Table 4 shows the seismic wave velocity measurement results in the worksheet of CM0002. This table includes the derived shear wave velocities (V_s_) and compressional wave velocities (V_p_) obtained using different methods. Note that the V_s_ measurements obtained by the cross-hole method have two records for each measurement depth. The reason for this is that the V_s_ measurements by the cross-hole method were cross-validated by exchanging receivers and sources, thus generating two different survey paths at the same depth, further enhancing their reliability. Fig. 3 shows the profiles of the V_s_ measurements using different methods at site CM as an example.

**Table 1:** Abbreviation of the sites and their coordinates.

Site label	Coordinate		Layout index (m)				
	longitude (E)	latitude(N)	A	B	C	D	E
NH	312625	2772594	1.07	1.01	2.64	2.90	1.00
KL	307504	2772480	1.08	1.37	2.79	2.73	1.28
GS	307434	2773646	1.04	1.03	2.75	2.86	1.02
MS	307877	2771882	1.04	1.18	2.90	2.63	1.04
CM	309371	2772215	1.12	1.05	2.70	2.22	1.22
MT	306181	2774378	-	1.03	5.09		0.95
TD	305147	2767877	-	1.01	4.91		1.00
DJ	304625	2774077	-	1.34	4.89		1.08
BL	302040	2774972	-	1.07	5.18		1.30
SZ	301408	2776185	-	1.01	4.85		1.19
DH	301268	2774254	-	1.19	4.95		1.29
YP	301075	2771811	-	1.04	4.86		1.16
HJ	299329	2769614	-	1.24	4.91		1.18

**Table 2:** Data example of SPT.

Sample	Rang of depth (m)		N	PI	Unit weight (kN/m^3^)	USCS	Soil content				Energy ratio(%)
							Grav.	Sand	Silt	Clay	
S-1	1.05	1.50	13	NP	16.5	SM	18	45	27	10	
S-2	2.55	3.00	2	9	16.3	CL	2	43	41	14	
S-3	4.05	4.50	4	11	17.8	CL	0	31	53	16	
S-4	5.55	6.00	1	NP	17.8	ML	0	47	37	16	
S-5	7.05	7.50	2	NP	19.9	ML	0	49	37	14	
S-6	8.55	9.00	3	13	19.0	ML	0	48	39	13	
S-7	10.05	10.50	4	22	18.6	CL	0	1	50	49	60
S-8	11.55	12.00	4	25	19.3	CH	1	2	52	45	61
S-9	13.05	13.50	4	31	19.5	CH	0	0	37	63	62
S-10	14.55	15.00	3	23	18.9	CL	0	1	41	58	67
S-11	16.05	16.50	4	24	18.2	CL	4	4	49	43	65
S-12	17.55	18.00	5	22	17.9	CL	0	10	52	38	65
S-13	19.05	19.50	6	17	17.4	CL	3	6	51	40	65
S-14	20.55	21.00	13	5	19.7	CL-ML	0	19	56	25	61
S-15	22.05	22.50	7	7	20.3	CL-ML	0	42	38	20	69
S-16	23.55	24.00	6	17	19.2	CL	0	26	49	25	73
S-17	25.05	25.50	6	14	18.8	CL	0	20	54	26	58
S-18	26.55	27.00	10	13	18.2	ML	0	26	51	23	61
S-19	28.05	28.50	18	4	18.1	CL-ML	0	37	46	17	60
S-20	30.00	30.45	30	NP	19.5	SM	0	84	12	4	63
GWL(m)											
4.1

**Table 3:** Data example of SCPT.

Depth (m)	q_c_ (MPa)	f_s_ (kPa)	u (kPa)	V_s_ (m/s)
0.05	0.50	4.10	0.09	0.00
0.10	1.27	10.23	-0.42	0.00
0.15	1.67	17.02	-0.62	0.00
0.20	2.10	23.15	-0.79	0.00
0.25	2.64	25.75	-0.94	0.00
0.30	3.05	27.54	-1.03	0.00
0.35	3.28	16.72	-1.06	0.00
0.40	3.37	21.90	-1.00	0.00
0.45	3.33	26.99	1.29	0.00
0.50	3.25	29.98	1.41	0.00
0.55	3.22	29.82	1.55	0.00
0.60	3.36	28.93	1.55	0.00
0.65	3.46	27.06	1.65	0.00
0.70	3.50	23.77	1.69	0.00
0.75	3.36	20.65	1.83	0.00
0.80	3.15	20.23	1.98	0.00
0.85	3.07	20.08	2.14	0.00
0.90	3.01	17.87	2.18	0.00
0.95	2.84	14.50	2.29	0.00
1.00	2.68	14.04	2.45	137.31
1.05	2.73	15.23	2.84	137.31
1.10	2.86	14.97	2.78	137.31
1.15	2.95	12.34	2.79	137.31
1.20	2.65	10.81	2.87	137.31
1.25	2.23	11.74	3.03	137.31
1.30	1.84	13.86	3.28	137.31
1.35	1.58	15.41	3.49	137.31
1.40	1.44	20.27	3.58	137.31
1.45	1.37	24.73	4.53	137.31
1.50	1.42	28.80	4.61	137.31
1.55	1.55	29.98	4.61	137.31
1.60	1.70	26.72	4.73	137.31
1.65	2.01	20.10	4.67	137.31
1.70	2.33	12.14	4.64	137.31
1.75	2.36	6.81	4.73	137.31
1.80	2.11	5.02	5.01	137.31
1.85	1.91	4.75	5.42	137.31
1.90	1.61	4.66	5.96	137.31
1.95	1.41	5.19	6.52	137.31
2.00	1.13	4.45	7.12	127.13

**Table 4:** Data example of Vs measurements.

Suspension PS-logging				Down-hole			Cross-hole			MASW	
Depth (m)	V_S_ (m/sec)	V_P_ (m/sec)	Poisson's ratio	Depth (m)	V_S_ (m/sec)	V_P_ (m/sec)	Depth (m)	V_s_ (m/sec)	V_s_ (m/sec)	Depth (m)	V_S_ (m/sec)
2.45	131.9	1369.9	0.495	0	140	491	0		120.7	0.0	150.1
3	126.3	1176.5	0.494	1	140	491	1	128.8	128.8	0.9	144.8
4	131.8	1388.9	0.495	2	140	491	2	128.5	128.3	2.0	144.7
5	135.3	1470.6	0.496	3	140	491	3	140.2	138.5	3.2	170.5
6	141.2	1515.2	0.496	4	140	491	4	138.9	135.5	4.4	173.1
7	135.5	1538.5	0.496	5	152	1873	5	136.7	135.6	5.8	162.6
8	138.1	1515.2	0.496	6	152	1873	6	137.0	138.0	7.3	158.7
9	147.7	1587.3	0.496	7	152	1873	7	137.7	138.0	8.8	167.3
10	158.0	1612.9	0.495	8	152	1873	8	140.6	139.7	10.5	176.0
11	164.7	1562.5	0.494	9	152	1873	9	148.3	149.2	12.3	199.3
12	165.0	1515.2	0.494	10	176	1873	10	161.4	158.5	14.2	204.0
13	172.7	1639.3	0.494	11	176	1873	11	163.9	163.0	16.2	219.2
14	187.3	1538.5	0.492	12	176	1873	12	168.6	169.7	18.3	218.0
15	201.6	1562.5	0.492	13	188	1873	13	175.1	178.6	20.5	215.9
16	204.5	1515.2	0.491	14	188	1873	14	184.6	182.8	22.8	233.2
17	225.7	1515.2	0.489	15	188	1873	15	190.4	191.5	25.3	232.0
18	233.1	1515.2	0.488	16	188	1873	16	199.3	199.3	27.8	231.6
19	221.7	1538.5	0.489	17	209	1873	17	219.6	215.1	30.4	232.0
20	236.4	1538.5	0.488	18	209	1873	18	223.5	226.4		
21	245.1	1694.9	0.489	19	209	1873	19	231.2	230.6		
22	230.4	1538.5	0.489	20	209	1873	20	235.8	231.6		
23	235.8	1639.3	0.489	21	209	1873	21	236.8	239.1		
24	251.9	1612.9	0.488	22	209	1873	22	250.3	244.9		
25	246.3	1666.7	0.489	23	235	1873	23	244.6	243.7		
25.3	243.9	1639.3	0.489	24	235	1873	24	244.8	241.4		
26	252.5	1562.5	0.487	25	235	1873	25	250.3	256.1		
26.6				26	235	1873	26	254.0	253.0		
28				27	235	1873	27	255.7	253.0		
29				28	256	1873	28	241.0	241.0		
30				29	256	1873	29	253.9	253.9		
31				30	256	1873	30	266.4	266.4

## 4 Experimental Design, Materials, and Methods

**Fig. 4:**
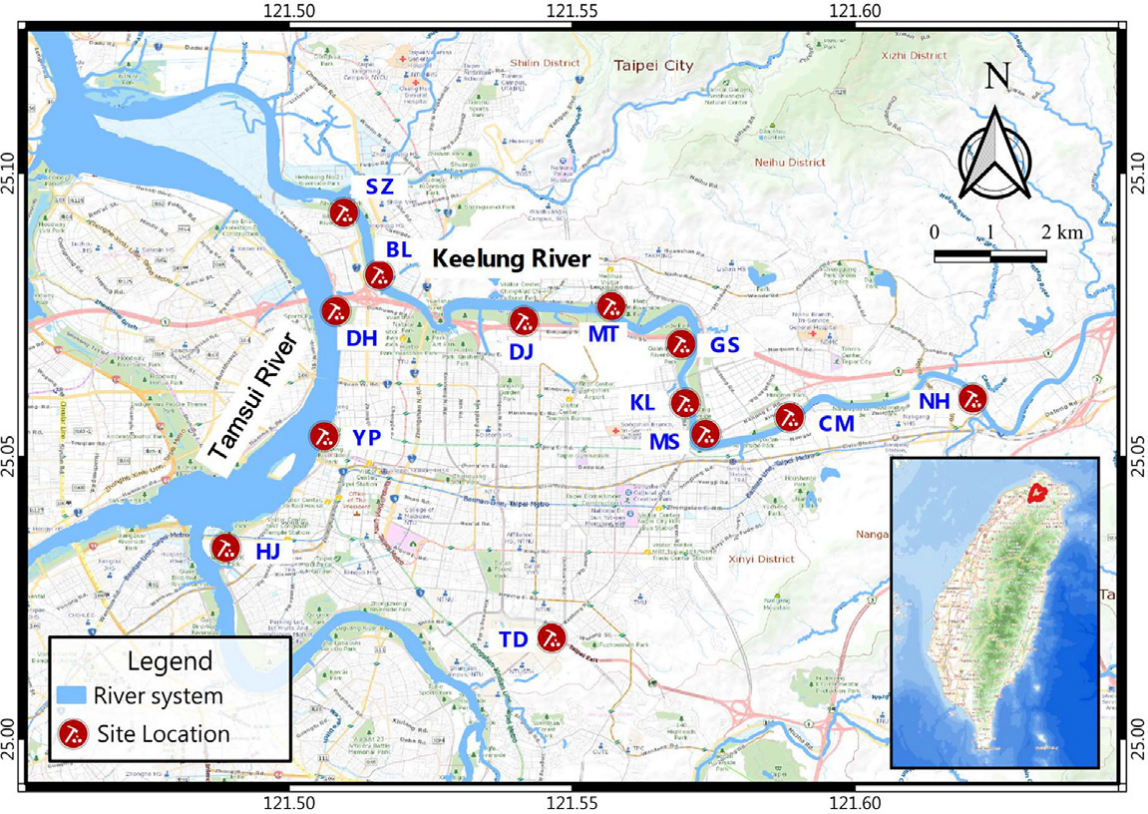
Locations of the thirteen test sites.

All tests were conducted over the period 27 December, 2018 to 9 April, 2019. All tests including SPT, SCPT, and a series of *in-situ* V_s_ measurements were meticulously planned at 13 selected Holocene alluvium sites in the Taipei Basin, with a primary focus on the Song-Shan layer. This layer is the youngest Holocene alluvium in the Taipei Basin and consists of interbedded sands, clays, and silts. To reduce interference from environmental vibrations, and to avoid underground pipeline obstacles during geological drilling, the selected testing sites were primarily chosen along banks of the Keelung and Tamsui Rivers. A map of the site locations is shown in Fig. 4.

### 4.1 Methods and standards

Fig. 2 displays the typical layout of the boreholes and soundings at each site. At each of the thirteen sites, two boreholes (BH-1 and BH-2) were drilled in accordance with international standard [1] and drilled by automatic and donut hammers, respectively. The two boreholes were cased with PVC tubes and backfilled with different materials. BH-1 was filled with pervious sandy gravel with an average grain size of 1 cm, while BH-2 was filled with cement–bentonite grout (with a bentonite to cement and water weight ratio of 1:2:15). These two materials are commonly adopted for backfilling after drilling. To better understand the influence of backfill and casing on V_s_ measurements, one extra borehole (BH-3), placed between the other two holes, was drilled at five of the 13 sites. At each site BH-3 was drilled with a donut hammer according to international standard [1] but it was not cased and backfilled until the suspension tests had been completed. While drilling the boreholes, energy ratio measurements were conducted in accordance with international standard [2]. Other than the boreholes, two SCPTs were conducted in accordance with international standard [3]. The corresponding borehole and SCPT were located within approximately 1 m of each other. Fig. 2 shows a typical layout of a site with three boreholes and two SCPT soundings.

The boreholes were used for traditional invasive seismic measurement (i.e., cross-hole, down-hole, and suspension methods). The depths of all the boreholes and SCPTs were approximately 30 m. The V_s_ measurement interval depth was 1 m, and measurements were made by professionals. To reduce potential interference due to drilling and effects of seismic tests on subsequent measurements, the time interval between drilling and the seismic testing was set to one week.

### 4.2 Data interpretation

In addition to these invasive seismic methods, non-invasive MASW tests were conducted at each site, covering the same horizontal ranges as the other seismic tests. The different seismic tests at each site were sufficiently close to one another such that measurements from these methods can be regarded as being conducted at an identical location. All seismic tests were carried out in accordance with international standards [4], [5], [6], [7], [8], except for MASW. For the MASW measurement, all test lines were over 60 m long and 23 geophones (4.5 Hz) placed at 3 m intervals were used to receive the signals generated by an active source in each line. The SeisImager/SW software was used to derive a dispersion curve by selecting the maximum amplitudes, and deterministic inversion of the dispersion curves was adopted. The MASW procedure was carried out according to the SeisImager manual [9].

**Fig. 5:**
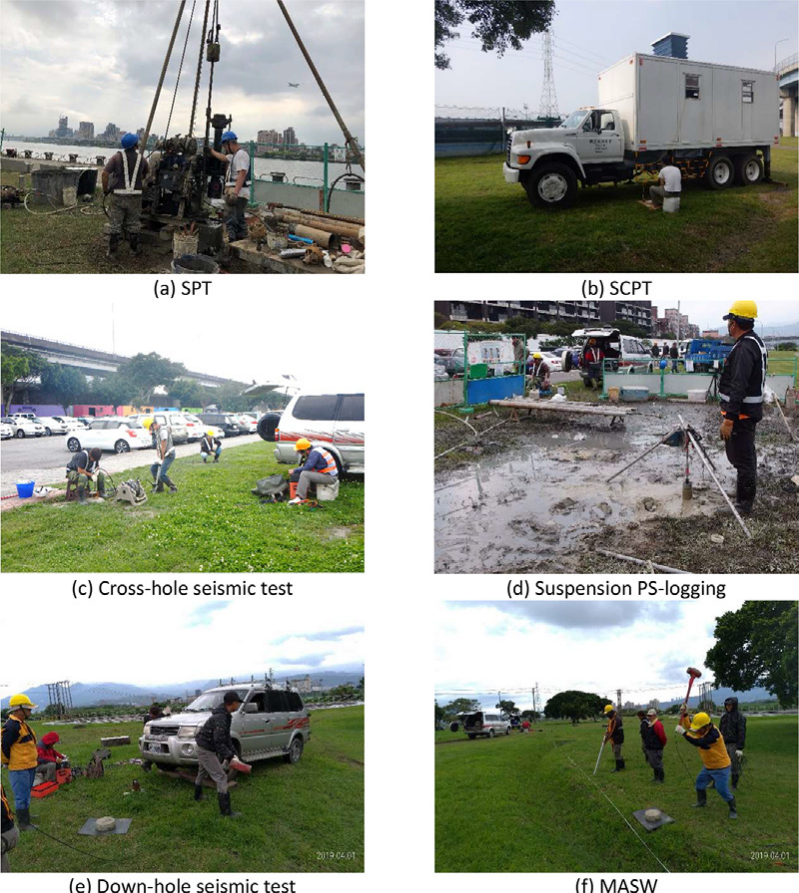
Photographs of methods employed at each test.

To ensure data quality, drilling and V_s_ measurement operations were supervised by professional engineers from the NCREE throughout the process, and experienced geotechnical experts irregularly inspected the implementation procedures. Moreover, the data processing procedure (e.g., picking arrival times) and the final measurement results were examined by a committee comprised of geotechnical engineering and geophysical science scholars and engineers from Taiwan. Fig. 5 shows photographs of supervision for each test.

## Limitations

Since the data were acquired in Holocene alluvium deposits in the Taipei Basin, the investigation produced data which are only representative of soft soils or low velocity geomaterials.

## Ethics Statement

The authors have read and follow the ethical requirements for publication in Data in Brief and confirming that the current work does not involve human subjects, animal experiments, or any data collected from social media platforms.

## CRediT authorship contribution statement

**Chih-Chieh Lu:** Conceptualization, Visualization, Writing – original draft, Project administration. **Yuan‑Chang Deng:** Supervision, Validation. **Jiun-Shiang Wang:** Data curation, Visualization, Writing – review & editing. **Jin-Hung Hwang:** Supervision, Validation. **Chun-Chieh Tseng:** Funding acquisition.

## Data Availability

Various geotechnical surveys in several arrays of Taipei Basin (Original data) (Mendeley Data).
